# Dynamic graph embedding for outlier detection on multiple meteorological time series

**DOI:** 10.1371/journal.pone.0247119

**Published:** 2021-02-18

**Authors:** Gen Li, Jason J. Jung

**Affiliations:** Department of Computer Engineering, Chung-Ang University, Dongjak-gu, Seoul, Republic of Korea; Western Norway University of Applied Sciences, NORWAY

## Abstract

Existing dynamic graph embedding-based outlier detection methods mainly focus on the evolution of graphs and ignore the similarities among them. To overcome this limitation for the effective detection of abnormal climatic events from meteorological time series, we proposed a dynamic graph embedding model based on graph proximity, called DynGPE. Climatic events are represented as a graph where each vertex indicates meteorological data and each edge indicates a spurious relationship between two meteorological time series that are not causally related. The graph proximity is described as the distance between two graphs. DynGPE can cluster similar climatic events in the embedding space. Abnormal climatic events are distant from most of the other events and can be detected using outlier detection methods. We conducted experiments by applying three outlier detection methods (i.e., isolation forest, local outlier factor, and box plot) to real meteorological data. The results showed that DynGPE achieves better results than the baseline by 44.3% on average in terms of the F-measure. Isolation forest provides the best performance and stability. It achieved higher results than the local outlier factor and box plot methods, namely, by 15.4% and 78.9% on average, respectively.

## 1 Introduction

Meteorological time series are part of climatic data and they have been extensively researched in many fields, including environmental science and computer engineering [1–3]. Outlier detection, which identifies instances that are distant from most other observations, is an important field of computer engineering and data mining. Outlier detection in meteorological time series is a necessary research issue because learning the patterns of abnormal climatic events can help reduce losses due to meteorological disasters [4, 5]. The concealed information obtained from meteorological data can be detected to analyze climatic changes.

Existing methods are mainly based on statistical indices [6] and machine learning algorithms, such as similarity-based methods [7] and density-based clustering methods [8]. These methods ignore the relationships among the time series, thus making it difficult to understand the causes of outliers. In a previous study [9], time-aware shapelets were extracted to construct an evolution graph to detect time series outliers. The approach was applied on a single signal, and outliers were detected only by comparing the signal with itself. This cannot explain the external factors affecting outliers. To solve this problem, we propose a method to discover the spurious relationship between two correlated time series that are not causally related, such that a dynamic graph can be constructed for detecting outliers.

Climatic events are denoted as a graph in which vertices indicate meteorological data and edges indicate the spurious relationship. The study by [10] identified four types of outliers in a dynamic graph, which are abnormal vertices, abnormal edges, abnormal subgraphs, and event detection. The outlier in the present study was a time interval in which the climatic event was abnormal, which is defined as follows.

**Definition 1 (Outlier in multiple time series)** *An outlier is defined as the time interval t*_*i*_ *in which the graph is significantly different from those in other time intervals, and it is formulated as P*(*t*_*i*_) < *θ*_1_ *or P*(*t*_*i*_) > *θ*_2_, *where t*_*i*_ *is the i*^*th*^ *time interval and P*(*t*_*i*_) *is a function used to calculate the score of t*_*i*_. *In addition, θ*_1_ *and θ*_2_ *are thresholds for detecting the outlier*.

**Fig 1:**
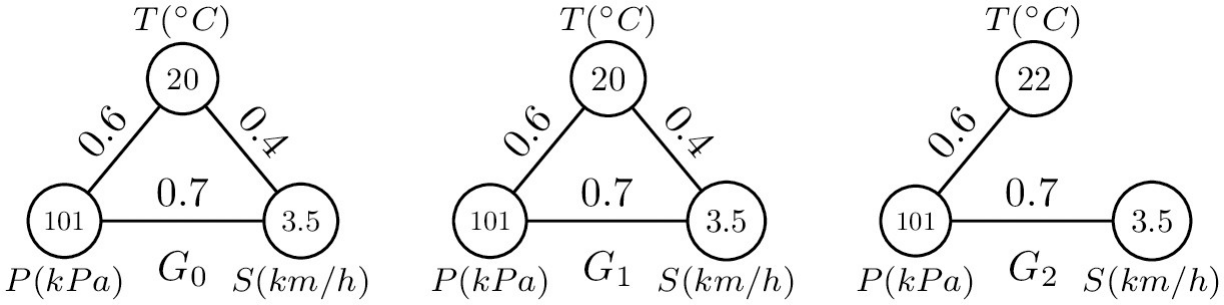
Example of the outlier.

Fig 1 shows an example of the outlier. There are three climate graphs at time interval *t* ∈ [0, 2], where *T* indicates the temperature, *P* indicates the pressure, *S* indicates the wind speed, and the edge indicates the spurious correlation. If there is an edge between two vertices, it indicates that there is a spurious correlation between two meteorological data. The edge between the temperature and speed at *t*_3_ is different from the other graphs, which leads the neighbor structure of the graph *G*_3_ is different so that the climatic event at *t*_3_ is abnormal. The time interval *t*_3_ is detected as an outlier.

To detect outliers, we used dynamic graph embedding, which uses a non-linear function to learn representation vectors of climatic events. Graph embedding has been applied in several fields to capture the information of nodes and edges to map a graph to a low-dimensional space. Existing methods, such as node clustering and link prediction, focus on issues entailing static graphs; consequently, temporal information is ignored. Because dynamic graphs record changes of graphs over a continuous period of time, the evolution of vertices and edges can be observed. Existing dynamic graph embedding models mainly focus on the evolution of graphs and ignore the similarities among them. To overcome this limitation, we propose a dynamic embedding model, called DynGPE. It learns the embedding vectors of dynamic graphs by exploiting graph proximity for clustering similar graphs.

The main contributions of this work are as follows.

- We propose DynGPE by improving structural deep network embedding (SDNE) [11]. The results show that DynGPE achieved the best performance in comparison with baselines.
- We conducted experiments on different real-world meteorological datasets by applying three outlier detection methods—isolation forest (IF) [12], local outlier factor (LOF) [13], and box plot [14]. The results indicate that the IF method achieved the best performance and stability based on DynGPE.

The remainder of this paper is organized as follows. In Sect. 2, studies related to outlier detection in time series are described. In Sect. 3, dynamic graph construction is detailed. In Sect. 4, the dynamic graph embedding model is described. In Sect. 5, the experimental results are presented. Finally, in Sect. 6, some concluding remarks regarding this study are provided.

## 2 Related work

In existing methods based on supervised detection, outliers are labeled in advance and a machine learning model learns the features from the outliers. Then, the model fits a non-linear function to detect outliers [15]. Su et al. [16] detected outliers by constructing a model that learns the robust representation for regular patterns of multiple time series. Outliers were detected based on the probability of reconstructing input data. The proposed model was applied on three real-world datasets, and it achieved better results than baselines. However, most time series are not labeled; therefore, many unsupervised learning methods have been proposed to address this issue [17, 18]. Autoencoder is an unsupervised learning model that has been widely applied to outlier detection on time series [19, 20]. Kieu et al. [21] constructed two recurrent autoencoders for outlier detection. Their model exhibited improved performance by avoiding overfitting. Yin et al. [22] highlighted the problem that an integration model constructed using an autoencoder and convolutional neural networks could not exhibit increased performance on time series data. To solve this problem, a recurrent neural network was added to the integration model. They conducted experiments on internet of things time series and achieved better performances than that of the baselines. These studies directly applied the models to the time series but ignored the hidden information among the multiple time series. DynGPE model constructs the dynamic graph by discovering the correlation between the data to solve this problem.

Four types of outliers in dynamic graphs have been highlighted in previous studies [23–25]. Our study mainly focuses on event detection. Several methods have been proposed previously to solve this problem. Because autoencoder performs well upon embedding, numerous studies have applied it to dynamic graphs. For example, Grattarola et al. [26] constructed two autoencoders to learn the representation vector of a dynamic graph for detecting changes. The results showed that the proposed method could identify small changes. Since the autoencoder model reconstructs the dynamic graph for calculating the embedding vectors, the proposed model ignores the temporal information. To solve this problem, Zhang et al. [27] proposed a temporal deep autoencoder architecture that considered the graph structure and vertex attributes to test the community. Ma et al. [28] proposed a community-aware dynamic network embedding method based on an autoencoder to record the dynamics of community structures. The results showed the proposed model performed well on existing graph issues (i.e., link prediction, network reconstruction). Leichtnam et al. [29] defined a security object graph and applied an autoencoder model to detect abnormal attacks from a network. These models have been considered the spatial and temporal information on the dynamic graph, but their similarity also needs to be considered. DynGPE model constructs the graph proximity to measure the similarity between two graphs to deal with the problem.

Graph-based outlier detection on time series aims to transform time series to graphs by discovering relationships. Boniol et al. [30] proposed a method for detecting outliers in domain agnostic time series in an unsupervised manner. They constructed a graph in which vertices are derived from overlapping trajectories and edges indicate transitions. The outliers in time series are detected by scoring the subsequence. Farag et al. [31] detected outliers in time series based on graphs. They used a slide window to scan time series and calculate distances among each subsequence. The graph was constructed using these distances, where the vertex indicates the subsequence and the weights of the edges are distances. The outliers were detected using a node clustering model. Gopalakrishnan et al. [32] analyzed the distributions of vertices on a dynamic graph and proposed a method to detect outliers in the dynamic graph. The method has been applied in airport networks to identify airplane delays. Walden et al. [33] constructed a brain functional connectivity group graph. Abnormal brain events were detected by calculating the frequency of electroencephalograms. These models utilized vertices to indicate the time series data and used the weights of edges to measure two vertices, such as distance. The reason of the outliers is that the correlation among the multiple time series is abnormal, so that DynGPE model discovers the spurious correlation to detect the outlier.

## 3 Dynamic graph construction

**Fig 2:**
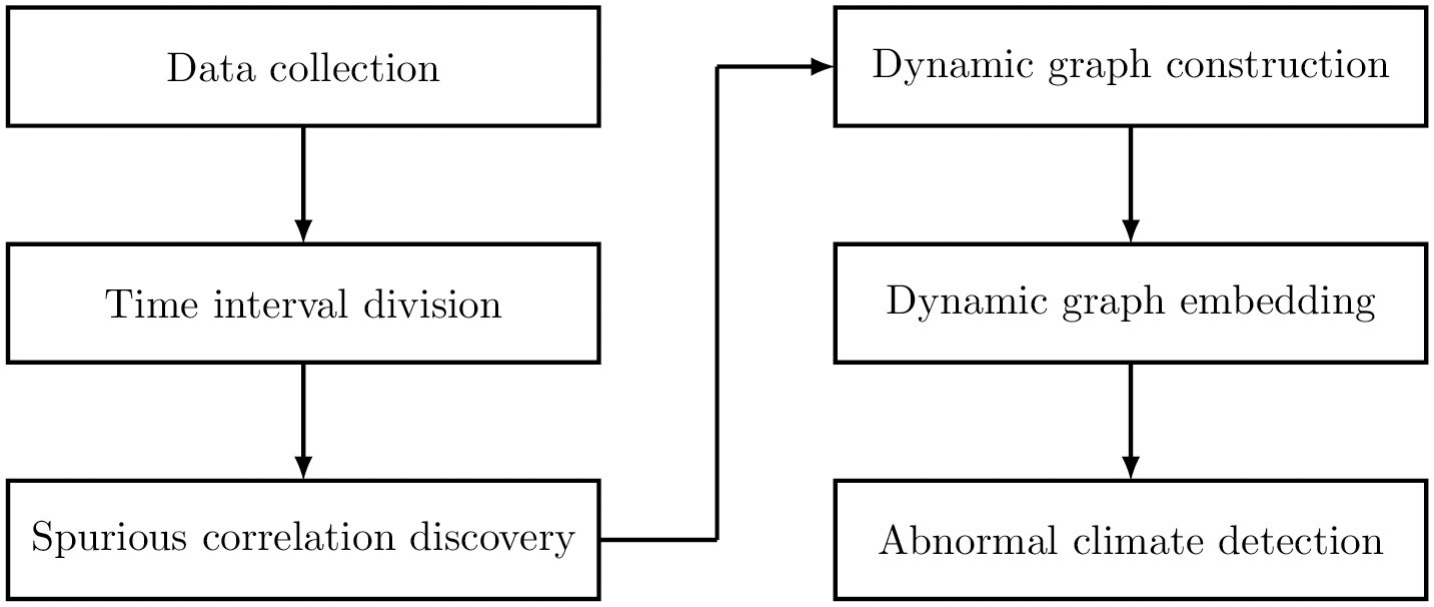
Architecture of the proposed method.

This section describes the construction of the dynamic graph for meteorological time series. As shown in Fig 2, the proposed approach includes six steps. Firstly, the real meteorological time series are collected from the China meteorological data service center by registering an account (http://data.cma.cn/en). Then, the study [34] proposed a time interval division method based on the wavelet transform by calculating the similarity between two time series. The spurious correlation is discovered based on causality and correlation. The dynamic graph is constructed by using the discovered spurious correlation where the vertex and edge indicate the meteorological data and spurious correlation, respectively. DynGPE is used to embed the dynamic graph for clustering climate events. Finally, the abnormal climate is detected by using the outlier detection methods.

In this study, we divide the time series into several time intervals. The short-run causality among the multiple meteorological data in a time interval needs to be obtained. Therefore, the Granger causality test [35], as the most general method for testing the short-run causality, was utilized. Pearson correlation coefficient [36] (PCC) can measure the linear correlation among two time series, so that it was used to discover the spurious relationship among time series. The short-run causality among the multiple time series can be defined as follows.

**Definition 2 (Short-run causality)** *Short-run causality indicates the causal relationship among the multiple time series in a short time interval, which is explained like that in this short time interval, one of the series is the cause of another series change, which is formulated as*

$$\begin{array}{c} C\left(x,y\right)=\{\begin{array}{ccc} 1 & if\;p<0.05 & \\ 0 & otherwise & \end{array} \end{array}$$ (1)

*where x and y are two time series, C*(*x*, *y*) *indicates the causality between them, and p is the probability that the two series are not causally related*.

We used the Ganger causality test to calculate the causality between two time series. We used one of the series as a variable to predict the other one and formulated a null hypothesis that the two time series are not causally related. The regression functions of the test are as follows.

$$\begin{array}{c} y_{t}=\sum_{j=1}^{q}\beta_{j}y_{t-j}+u_{1} \end{array}$$ (2)

$$\begin{array}{c} y_{t}=\sum_{i=1}^{q}\alpha_{i}x_{t-i}+\sum_{j=1}^{q}\beta_{j}y_{t-j}+u_{2} \end{array}$$ (3)

where *α* and *β* are coefficients of time series *x* and *y*, respectively. Variables *u*_1_ and *u*_2_ denote the noise, *q* is the lag length, and *t* is the time point. Eq 2 can be used to predict the current *y*_*t*_ using the past value of the series *y*. In Eq 3, the past value of time series *x* is used as a variable to predict *y*_*t*_. The test proposes that if series *x* is helpful in the prediction of *y*, the regression result of Eq 3 is better than that of Eq 2, and there is a causality between them. The t-test was utilized to infer the differences in results between the two functions [37]. The probability of the null hypothesis is denoted as *p*. If the *p* value is less than 0.05 [38], it indicates that the two series are causally related.

PCC is used to represent the correlation among time series, which is calculated using covariance and variance. The spurious relationship can be discovered using these two relationships. If PCC is zero, the two time series are not correlated. A spurious relationship can be defined as follows.

**Definition 3 (Spurious relationship)** *Two correlated time series that are not causally related are said to exhibit a spurious relationship; this can be formulated as follows*.

$$\begin{array}{c} R\left(x,y\right)=\{\begin{array}{ccc} 1 & if\;C(x,y)-|PCC|<0 & \\ 0 & otherwise & \end{array} \end{array}$$ (4)

*where R*(*x*, *y*) *indicates the spurious relationship between two time series x and y*. *If C*(*x*, *y*) − |*PCC*| *is less than 0, it indicates that there is no causality between the two series, and the relationship is spurious*.

A graph is denoted as *G* = (*V*, *E*), where *V* and *E* denote the vertices and edges, respectively. The weight value of an edge is denoted as *w*, which equals *R*(*x*, *y*). The adjacency matrix is composed of the neighbor structure of each vertex, which is denoted as *A*.

**Definition 4 (Dynamic graph)** *A dynamic graph is defined as a set that consists of graphs at each time interval t* ∈ [0, *T*], *which is denoted as* $G=\{G_{t}|t\in \left[0,T\right]\}$. *The dynamic adjacency matrix is composed of adjacency matrices, which are denoted as* $A=\{A_{t}|t\in \left[0,T\right]\}$.

The dynamic graph is constructed by utilizing the spurious relationships, where the graph indicates a climatic event, vertices indicate meteorological data, and the edges indicate spurious relationships.

## 4 Dynamic graph embedding

Graph embedding involves yielding a graph *G* = (*V*, *E*) with |*V*| = *N* and learning a map function *f*: *V*_*i*_ → *v*_*i*_, where *v*_*i*_ is an embedding vector of the vertex *V*_*i*_ and *N* is the number of vertices. Our task is learning the representation of the dynamic graph G, which is to learn a map function *f*: *G*_*t*_ → *g*_*t*_, where *g*_*t*_ is an embedding vector of the graph *G*_*t*_ at each time interval *t* ∈ [0, *T*].

The DynGPE model is proposed by improving the SDNE model which defines two proximities. The first-order proximity preserves the global graph structure, which indicates that two vertices with an edge have a short distance in an embedding space. The second-order proximity preserves the local graph structure, which reduces the loss between the input vector and the reconstructed vector. To maintain similar graphs in a short distance in a feature space, we modify the first-order proximity and provide a definition of graph proximity, which is as follows.

**Definition 5 (Graph proximity)** *Graph proximity is defined as the similarity between two graphs on the neighbor structure*. *Consider that A*_*i*_ *and A*_*j*_ *are adjacency matrices of the graphs G*_*i*_ *and G*_*j*_, *respectively*. *The graph proximity between G*_*i*_ *to G*_*j*_ *is formulated as* $d\left(A_{i},A_{j}\right)=\sqrt{\left|\right|A_{i}-A_{j}{\left|\right|}_{2}^{2}}$ *for measuring the similarity between G*_*i*_ *to G*_*j*_ *on the neighbor structure*.

**Fig 3:**
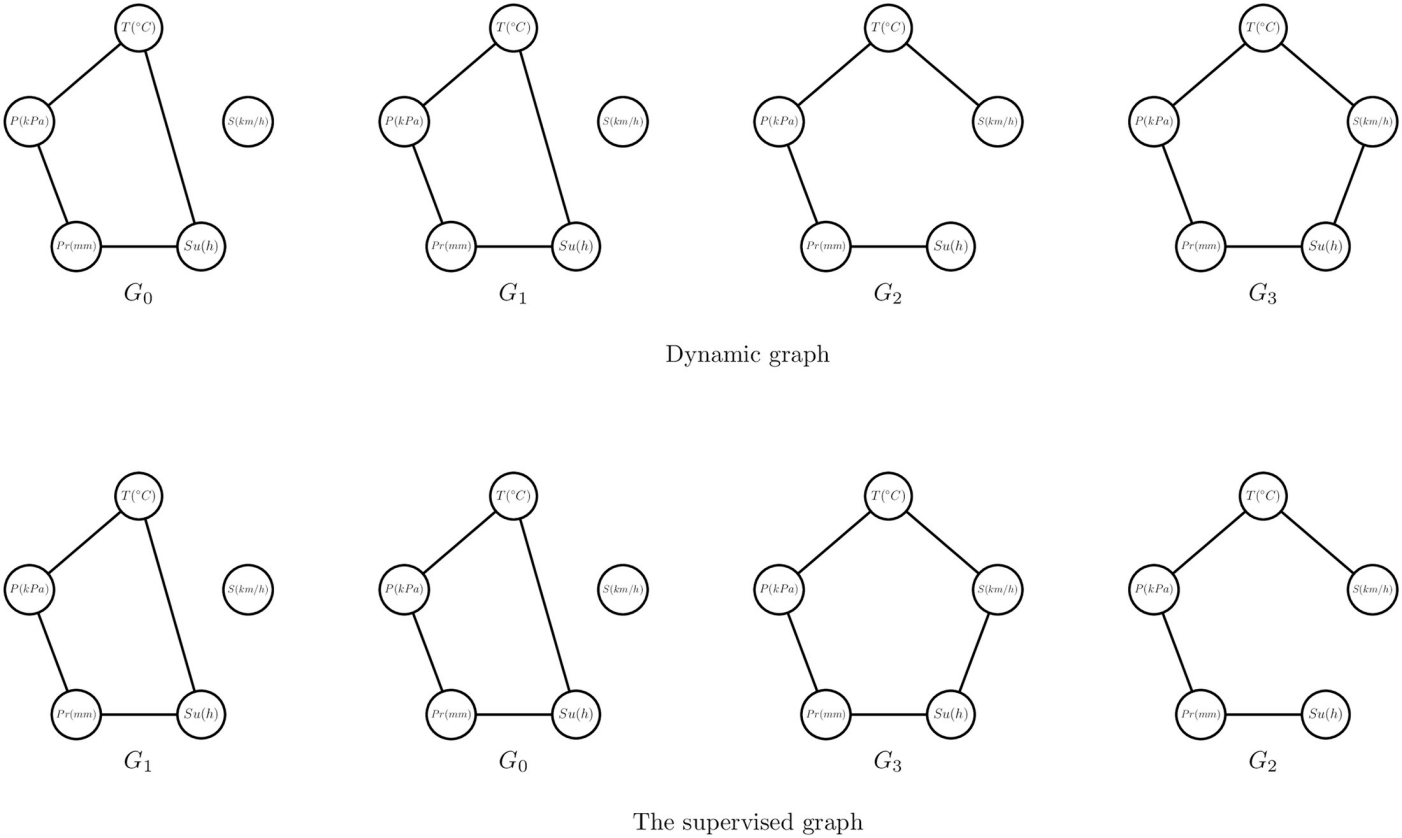
Example of the dynamic supervised matrix.

As shown in Fig 3, the dynamic graph is formulated as $G=\langle G_{0},G_{1},G_{2},G_{3}\rangle$, where each graph *G*_*i*_ indicates one climatic event, *T* indicates the temperature, *P* indicates the pressure, *S* indicates the wind speed, *Pr* indicates the precipitation, and *Su* indicates the sunlit time. The dynamic adjacency matrix is formulated as $A=\langle A_{0},A_{1},A_{2},A_{3}\rangle$. The graph proximity between *G*_0_ and *G*_1_ is calculated as *d*(*A*_0_, *A*_1_) = 0, so that the nearest graph from *G*_0_ is *G*_1_. In this way, the nearest graph from *G*_1_ is *G*_0_, the nearest graph from *G*_2_ is *G*_3_, and the nearest graph from *G*_3_ is *G*_2_. Therefore, the set of these nearest graphs is formulated as *S* = 〈*G*_1_, *G*_0_, *G*_3_, *G*_2_〉. The dynamic supervised graph is constructed to record the nearest graphs and is defined as follows.

**Definition 6 (Dynamic supervised graph)** *Let* $G=\{G_{t}|t\in \left[0,T\right]\}$ *denote the dynamic graph at time interval t* ∈ [0, *T*]. *For the graph G*_*t*_, *the corresponding G*_*i*_ *can be found from the dynamic graph* G, *where the G*_*i*_ *is the nearest graph of the G*_*t*_. *The dynamic supervised graph is a set composed of the graph G*_*i*_, *which is formulated as* $S=\{G_{i}|i\in \left[0,T\right]\}$.

**Fig 4:**
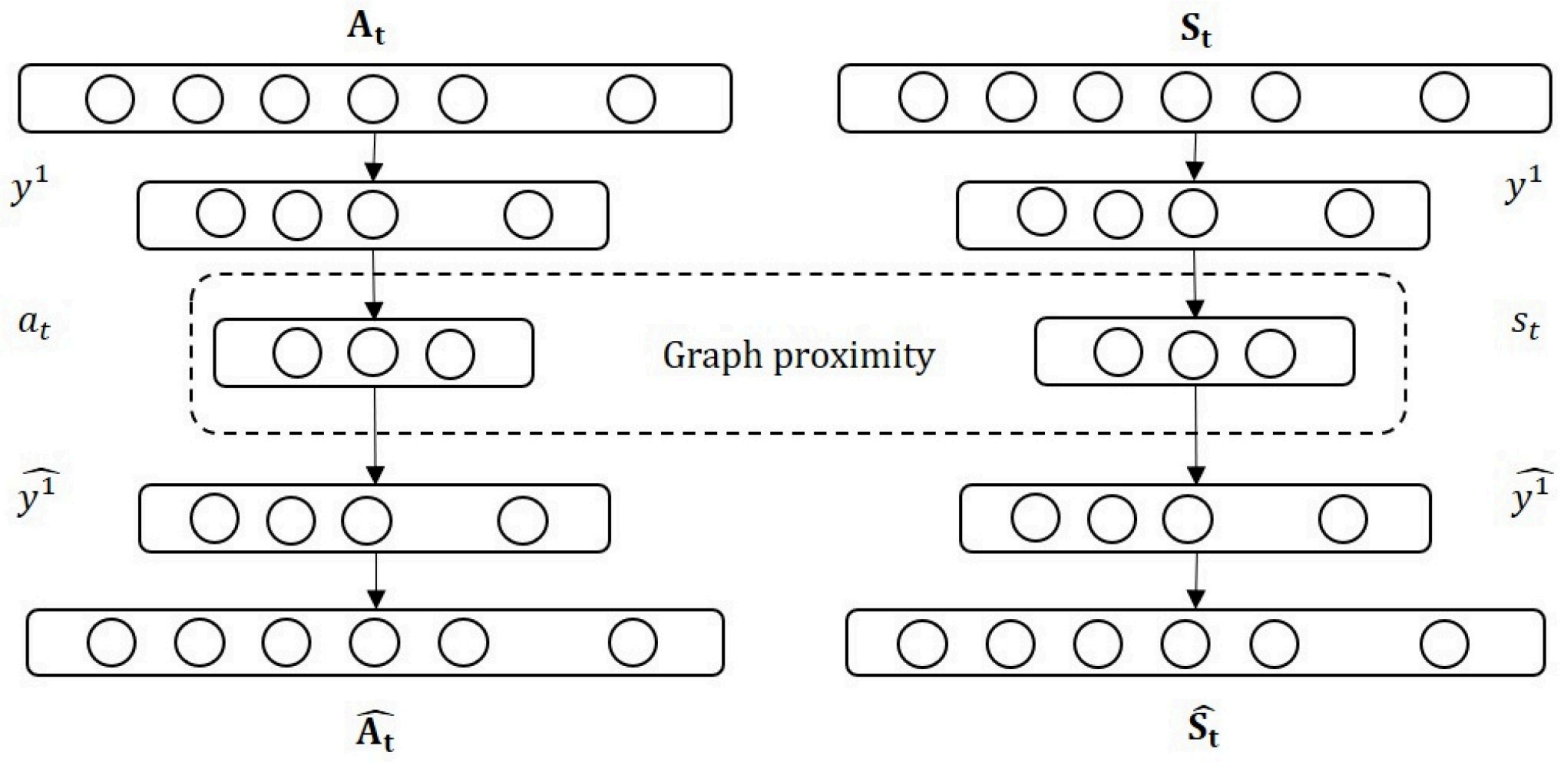
Architecture of DynGPE model.

The adjacency matrix and supervised matrix are denoted as *A*_*t*_ and *S*_*t*_, respectively. The architecture of DynGPE is shown as Fig 4, where *t* ∈ [0, *T*] indicates the number of time intervals. In this model, the embedding vectors of *A*_*t*_ and *S*_*t*_ are denoted as *a*_*t*_ and *s*_*t*_, respectively. The model was constructed using an autoencoder which consists of an encoder and a decoder. The *i*^*th*^ layers of the encoder and decoder are denoted as *y*^*i*^ and $\hat{y^{i}}$, respectively. The outputs of the two decoders are $\hat{A_{t}}$ and $\hat{S_{t}}$.

The encoder maps the input vector to the embedding space using a non-linear function. Given input *A*_*t*_, the output of each hidden layer in the encoder is shown as follows.

$$\begin{array}{c} y^{1}=\delta \left(W^{1}A_{t}+b^{1}\right) \end{array}$$ (5)

$$\begin{array}{c} y^{i}=\delta \left(W^{i}y^{i-1}+b^{i}\right) \end{array}$$ (6)

where *i* ∈ [2, *I*] indicates the number of layers, and *δ* indicates the ReLU function that is one of the activation function in the neural network for making the neural network non-linear [39]. Relu function can be formulated as *f*(*y*^*i*^) = *max*(0, *y*^*i*^). The weight and basis of the *i*^*th*^ layer are denoted as *W*^*i*^ and *b*^*i*^, respectively. The decoder $\hat{y^{i}}$ can be calculated by reversing the calculation of the encoder. The output of *y*^*I*^ is the embedding vector *a*_*t*_ of graph *G*_*t*_.

For loss functions, because the size of the dynamic adjacency matrix is not large and most elements are not zeros, we move the penalty items from the loss functions. Two loss functions $L_{1}=\frac{1}{T}\sum_{t=1}^{T}\left|\right|a_{t}-s_{t}{\left|\right|}_{2}^{2}$ and $L_{2}=\frac{1}{T}\sum_{t=1}^{T}\left|\right|A_{t}-\hat{A_{t}}{\left|\right|}_{2}^{2}$ are established for two proximities. $L_{1}$ can be used to develop similar graphs to achieve a short distance in an embedding space, and $L_{2}$ maintains the similarity between the input graph and reconstructed graph. For the optimizing model, we establish a joint loss function, which is formulated as follows.

$$\begin{array}{c} L=\frac{1}{T}\sum_{t=1}^{T}\left|\right|A_{t}-\hat{A_{t}}{\left|\right|}_{2}^{2}+\frac{1}{T}\sum_{t=1}^{T}\left|\right|a_{t}-s_{t}{\left|\right|}_{2}^{2}+L_{reg} \end{array}$$ (7)

$$\begin{array}{c} L_{reg}=\frac{1}{2}\sum_{i=0}^{I}\left(|\right|W^{i}{\left|\right|}_{2}^{2}+\left|\right|\hat{W^{i}}{\left|\right|}_{2}^{2}) \end{array}$$ (8)

where *W*^*i*^ and $\hat{W^{i}}$ indicate the weights of the *i*^*th*^ layer in the encoder and decoder, respectively. Eq 8 presents the regularization term used to avoid overfitting.

## 5 Experimental results

In this section, we applied DynGPE using Python 3.7 with the NumPy, Pandas, and torch libraries. For optimizing DynGPE, we utilized the Adam optimizer to update and calculate the weights [40].

### 5.1 Dataset

To launch DynGPE, we extracted daily climatic data from Chinese surface stations of five cities, which are Beijing, Shanghai, Guangdong, Shandong, and Shanxi. Each time series was collected from 1990 to 2020. Further, the meteorological data of each city include 18 time series (e.g., pressure, temperature, humidity, precipitation, sunshine duration, water vapor and so on).

### 5.2 Evaluation metric

To the best of our knowledge, there is no ground truth data in the datasets. Thus, to validate DynGPE, we label some outliers in each dataset as follows. We assume that there are 10% outliers in each dataset and the embedding vector of the *t*^*th*^ graph is denoted as *e*_*t*_. The center of embedding vectors is formulated as $c=\frac{1}{T}\sum_{t=0}^{T}e_{t}$, where *t* indicates the number of time intervals. Similar graphs have a short distance in an embedding space using DynGPE. If most climatic events are similar, their embedding vectors are close to the center, and abnormal climatic events are far from the center. Based on this hypothesis, we labeled 10% embedding vectors that are at the farthest distance from the center as outliers.

### 5.3 Results and analysis

The first baseline model employed here is the graph convolutional neural (GCN) [41], which utilizes a convolution kernel to extract the information regarding vertices and edges. It does not include the temporal information of graphs. The second one is based on the architecture of dyngraph2vec [42], which is an unsupervised model for learning the representation of dynamic graphs. It provides four architectures, which are dyngraph2vecAE, dyngraph2vecRNN, and dyngraph2vecAERNN for evaluating DynGPE.

Table 1 exhibits the performance of using the IF method under 10% outliers. According to the results, DynGPE achieves the best result for each city because GCN and dynagraph2vecAE only capture information from a single graph; moreover, dyngraph2vecRNN only captures the temporal information of the dynamic graph but ignores the similarities of the graph. All models performed the best for the dataset of Beijing. This indicates the most normal climatic events in Beijing are similar and the outliers can be easily observed. All four models performed the worst for Shanxi. This indicates that the similarities among normal climatic events in Shanxi are not high and most of the events are isolated. IF detected them as outliers; therefore, the corresponding performance was lower than that in the cases of the other cities.

**Table 1:** Comparison experiments by using IF.

	Beijing	Shanghai	Guangzhou	Shandong	Shanxi
GCN	0.615	0.526	0.512	0.419	0.571
dyngraph2vecAE	0.617	0.421	0.503	0.638	0.535
dyngra ph2vecRNN	0.535	0.408	0.479	0.553	0.438
dyngraph2vecAERNN	0.520	0.435	0.476	0.480	0.476
DynGPE	**0.909**	**0.762**	**0.727**	**0.800**	**0.714**

Table 2 shows the performance of DynGPE with 10% outliers. According to the results, the IF method performed the best for the four cases. The performance for Beijing was better than that for other cities using LOF and IF. This indicates that the climatic events of Beijing at the feature space are more centralized than those of other cities. The performance of LOF for Guangzhou was better than that of IF, but the performance of IF was lower than that of LOF only by 0.019. The performance of the box plot method was lower than that of the other two methods. Overall, the results indicate that IF exhibits the highest performance on more cases using DynGPE, and the box plot method exhibits the worst performance.

**Table 2:** Performance of DynGPE.

City	LOF	IF	Box-plot
Beijing	0.788	**0.909**	0.528
Shanghai	0.727	**0.762**	0.667
Guangzhou	**0.741**	0.727	0.683
Shanxi	0.706	**0.800**	0.533
Shandong	0.690	**0.714**	0.488

**Fig 5:**
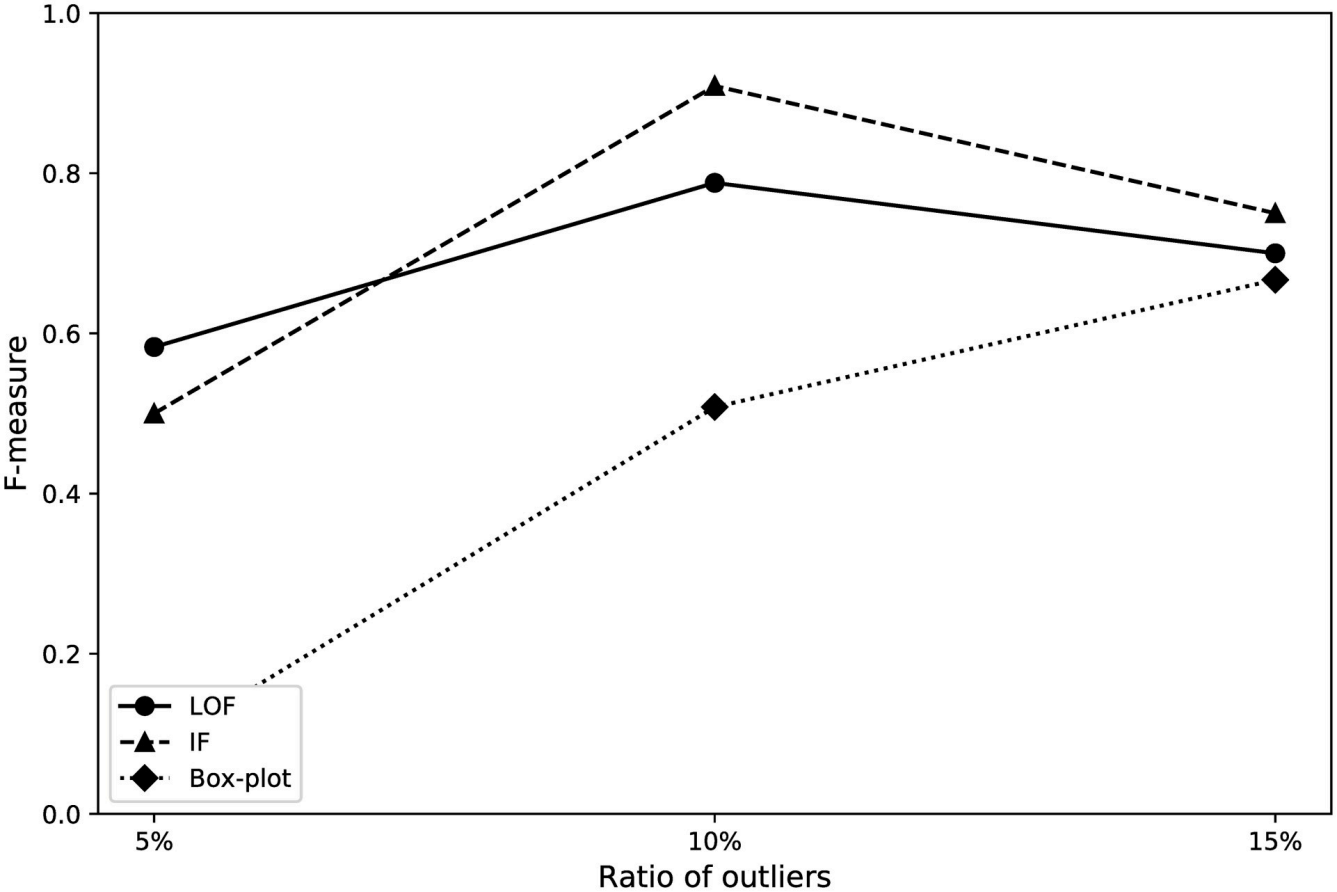
Performance for the city of Beijing.

Fig 5 shows the performance with different ratios of outliers for Beijing. According to the results, IF performed the best in terms of the F-measure for 2 cases; particularly, the best performance, corresponding to an F-measure of 0.909, was obtained for 10% outliers. LOF achieved the best F-measure on 5% outliers (the result was 0.583). Overall, the results indicate that the box plot method achieved the worst F-measure among all methods because it is based on the statistical method. The distribution of embedding vectors is discrete; therefore, the outlier detection performance is not better than that of other methods. The performance of IF is less than that of LOF for 5% outliers. If there are 5% outliers in the dataset, the climatic events between outliers and inliers are also relatively isolated, such that IF becomes prone to mistakenly detecting these points as outliers.

**Fig 6:**
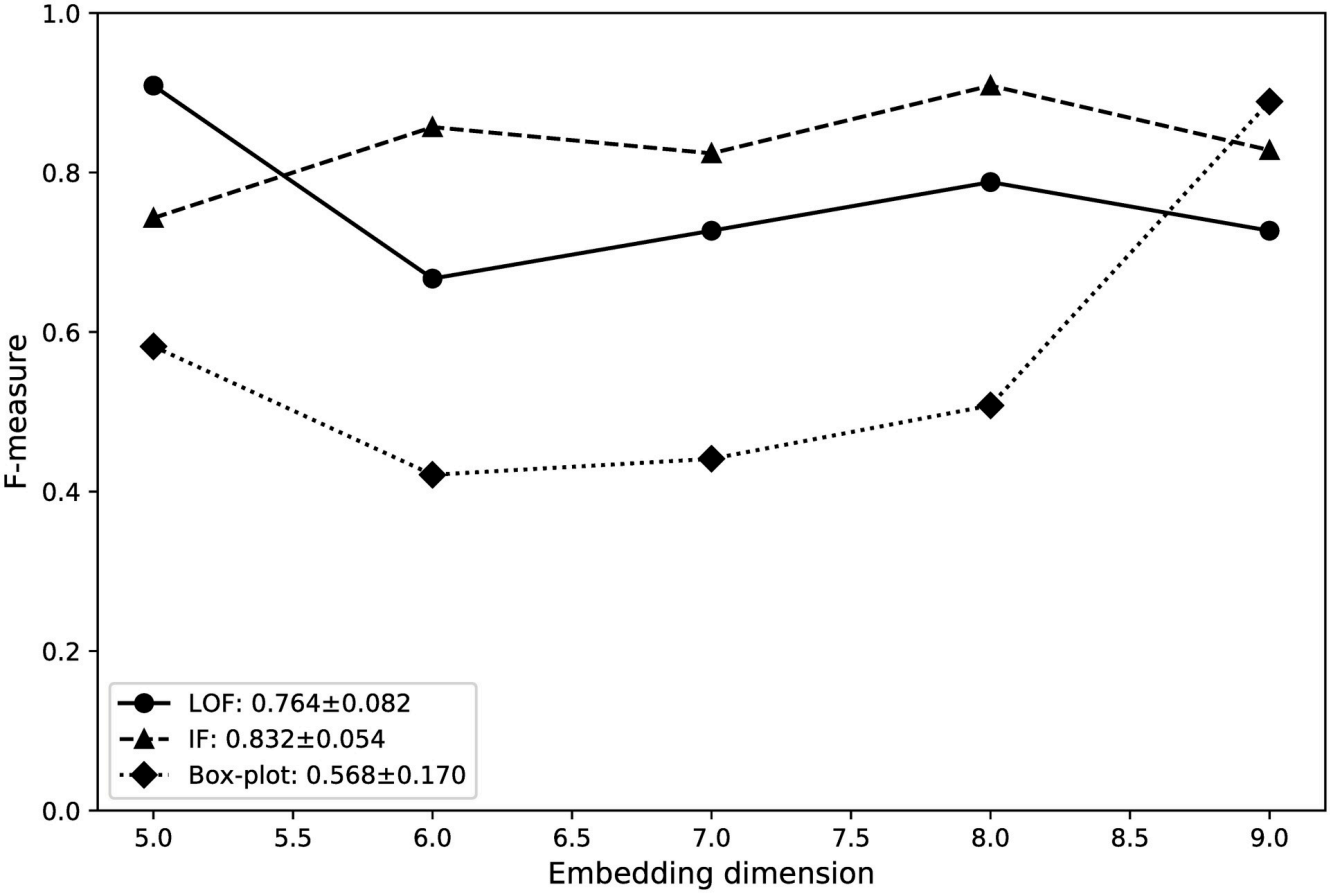
Evaluation of stability.

We analyzed the stability of DynGPE. Fig 6 shows the performance under different embedding dimensions. The stability of DynGPE was evaluated using *mean*±*std*, where *std* indicates the standard deviation, to measure the dispersion of results. According to the results, IF performed the best and was the most stable compared with other methods; further, it achieved the highest result for the embedding dimension of 8 with an F-measure of 0.909. Among all the methods employed in this study, the performance of the box plot method was the worst, and its stability was worse than that of other methods.

Overall, the evaluation results indicate that IF can achieve the best performance and stability with DynGPE for outlier detection. Among the climatic data extracted from five cities, IF achieved the best results for four cities. When there are 10% outliers in datasets, IF achieves higher results than the LOF and box plot methods. In terms of stability, the performance of IF is better than that of other methods. In this experiment, IF not only achieves the best F-measure on the average embedding dimension but also has the minimum standard deviation. In contrast, the box plot method showed the worst performance in all evaluation experiments because the box plot detects outliers based on the distribution of data points. If the data do not follow a Gaussian distribution, the performance of the box plot method is considerably reduced. The comparison results obtained using different models indicated that DynGPE performs better than the baselines.

## 6 Conclusion

In this paper, we propose DynGPE for detecting abnormal climatic events using meteorological data. It utilizes dynamic autoencoders to capture the information of graphs for reducing their distance from similar graphs. DynGPE constructs a dynamic supervised matrix to yield the graph proximity of the dynamic graph. Our experiments verify the performance and stability of different methods for outlier detection based on DynGPE. IF exhibits the best performance and stability for outlier detection and achieves higher results than the LOF and box plot methods, namely, by 15.4% and 78.9%, respectively. The experimental results show that DynGPE performs better than other graph embedding models and achieves results that are higher than those obtained by the other methods by 44.3% on average. The experimental results showed that the climatic events of Beijing are stable because most graphs are relatively concentrated in the embedding space. This indicates that most climate events are similar, and outliers can be easily detected, because of which the performance for Beijing is the best.

There are two limitations in this study. The first is that DynGPE is based on an autoencoder that captures the global information of graphs to detect an event from a dynamic graph. It ignores temporal information and cannot detect changes in the dynamic graph. To overcome this issue, we plan to combine a graph convolutional network and autoencoder to construct a dynamic graph embedding model for detecting events and changes from a dynamic graph. The second is that the paper considers the short-run causality and linear correlation but ignores the immediate causality and rank correlation coefficient. To solve this problem, we plan to use advanced causality measurement techniques such as the Geweke test [43] and the Spearman’s rank correlation coefficient [44] in future work.
